# New-Onset Atrial Fibrillation in COVID-19 Infection: A Case Report and Review of Literature

**DOI:** 10.7759/cureus.23912

**Published:** 2022-04-07

**Authors:** Deesha Shah, Zaryab Umar, Usman Ilyas, Nso Nso, Milana Zirkiyeva, Vincent Rizzo

**Affiliations:** 1 Internal Medicine, Icahn School of Medicine at Mount Sinai, Queens Hospital Center, New York City, USA

**Keywords:** covid 19, atrial fibrillation, covid-19 cardiovascular complications, arrhythmia, covid-19 infection, new onset atrial fibrillation

## Abstract

Since the beginning of the coronavirus disease 2019 (COVID-19) pandemic, many cases of arrhythmias have been reported in patients with COVID-19 infection. We present the case of a 66-year-old female with no known cardiovascular history who presented with worsening shortness of breath and productive cough and tested positive for COVID-19 infection in the ED. The patient had a recent hospitalization for COVID-19 infection during which she was treated with dexamethasone and remdesivir therapy and her course remained uncomplicated at that time. Following this, she developed worsening shortness of breath at home for which she presented to the ED. During this hospitalization, she was treated with dexamethasone, remdesivir, and supplemental oxygen. On day six of hospitalization, the patient became tachycardic and had palpitations. Cardiac monitor and EKG showed evidence of new-onset atrial fibrillation (NOAF). Initially patient received metoprolol and diltiazem, both of which failed to achieve adequate rate control. Following this, the patient was started on carvedilol 30 mg every six hours, which attained good rate control. Her CHA2DS2-VASc (congestive heart failure, hypertension, age ≥75 (doubled), diabetes, stroke (doubled), vascular disease, age 65 to 74, and sex category) score was 4 for which she was started on apixaban 5mg twice daily. The patient was discharged on the same medications. Despite increasing reported incidences of NOAF in COVID-19 infection, only little is known about the optimal management strategies and possible etiopathology. The aim of our review is to highlight the possible mechanisms triggering atrial fibrillation in COVID-19 infection and go over the management strategies while reviewing the available literature.

## Introduction

The severe acute respiratory syndrome coronavirus 2 (SARS-CoV-2) potentiates the highly contagious viral infection that leads to the development of coronavirus disease 2019 (COVID-19) [1]. The cardiovascular complications of COVID-19 have caused a global health crisis that resulted in millions of deaths since the onset of the pandemic situation. COVID-19 predominantly impacts cardiovascular function by triggering cytokine release syndrome that progresses to a systemic inflammatory response with the potential to deteriorate cardiac myocytes and vascular endothelium. It also triggers acute respiratory distress syndrome resulting in hypoxia, thereby elevating the cardiometabolic demand. The viral myocarditis and acute injury to endothelial cells, heart, and lungs aggravate further by the consistent interaction between angiotensin-converting enzyme 2 (ACE-2) receptors and viral antigen [2]. The potential cardiovascular complications of COVID-19 include myocardial infarction, stroke, and arrhythmias that add to the incidence of morbidity and cardiac deaths. Atrial fibrillation is a highly prevalent arrhythmic condition triggered by COVID-19 infection [3]. The atrial fibrillation triggered by infections worsens the prognosis by aggravating the structural heart disease; however, the severity of infections determines the prognostic outcomes [4]. The new-onset atrial fibrillation (NOAF) predicts the worst cardiovascular outcomes in the setting of COVID-19 [5]. The contemporary literature lacks data to evaluate the progression and outcomes of NOAF in COVID-19 patients. This case report with a literature review provides current data on NOAF in the context of COVID-19. It also elaborates on NOAF complications and management strategies in COVID-19 scenarios. 

## Case presentation

**Figure 1:**
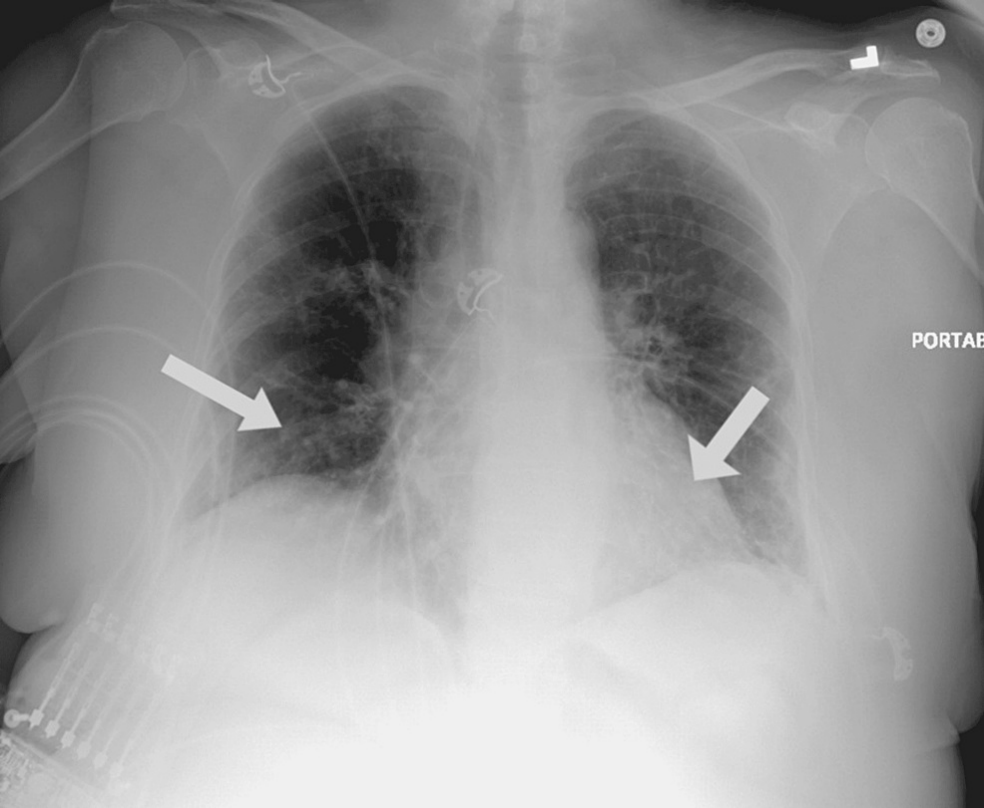
Chest x-ray: bilateral patchy opacities (white arrows) consistent with COVID-19 pneumonia. COVID-19: coronavirus disease 2019

**Figure 2:**
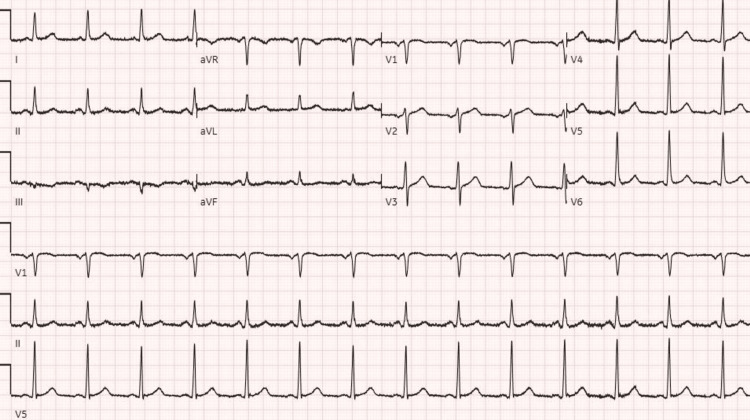
EKG on the day of admission showed normal sinus rhythm at a rate of 84 beats per minute.

**Figure 3:**
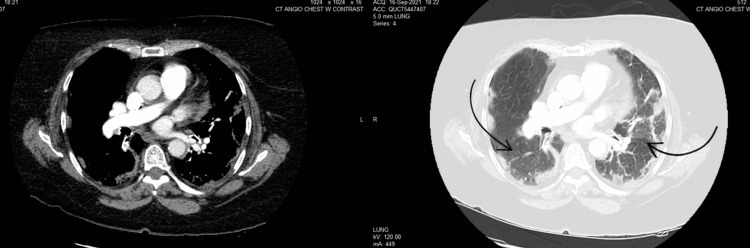
CT angiogram: bilateral multifocal pneumonia (black arrows) with no evidence of acute pulmonary embolism.

We present a 66-year-old female with a past medical history of asthma, non-insulin-dependent type 2 diabetes mellitus, and hypertension presented to the emergency department with worsening shortness of breath and productive cough with white sputum one day after a recent discharge during which she was treated for acute hypoxic respiratory failure secondary to COVID-19 infection. The patient was treated with five days of dexamethasone and remdesivir after which she was discharged without supplemental oxygen. During the above-mentioned admission, the patient required 3L of oxygen via nasal cannula and required no supplemental oxygen upon discharge. On this presentation, the patient was saturating at 81% on room air, tachycardic to 106/min, and had bilateral expiratory wheezing. Chest x-ray showed bilateral mid to lower lung hazy opacities consistent with COVID-19 pneumonia (Figure 1). EKG showed normal sinus rhythm with 85 beats per minute (Figure 2). D-dimer was elevated to 363 (Table 1). The patient was put on a 5L nasal cannula saturating to 95% and was started on remdesivir 100mg daily and dexamethasone 6mg daily. The patient was not loaded with remdesivir as she received it during her previous hospitalization. In addition, the patient received Xopenex and ipratropium inhalation therapy for asthma exacerbation. A lower extremity duplex was done, which was negative for any thrombus. CT angiogram of the chest was done with evidence of bilateral multifocal pneumonia consistent with COVID-19 infection and no evidence of pulmonary embolism (Figure 3).

**Table 1:** Laboratory results on the day of admission. BUN: blood urea nitrogen; ALK PHOS: Alkaline phosphatase; ALT: alanine transaminase; AST: aspartate aminotransferase; TSH: thyroid-stimulating hormone; IL-6: Interleukin 6; COVID-19: coronavirus disease 2019; PCR: polymerase chain reaction; DDU: D-dimer unit

Labs results on the day of admission	Value	Reference range and units
Hemoglobin	12.2	11.5-15.5 g/dL
Hematocrit	36.9	34.5-45.0%
WBC	10.83	3.80-10.50 K/uL
Sodium	124	135-145 mmol/L
Potassium	3.5	3.5-5.3 mmol/L
BUN	7	7-23 mg/dL
Creatinine	0.56	0.50-1.30 MG/dL
Albumin	3.7	3.3-5.0 g/dL
Total protein	6.7	6.0-8.3 g/dL
Total Bilirubin	0.4	0.2-1.2 mg/dL
ALK PHOS	68	40-120 U/L
ALT	28	10-45 U/L
AST	20	10-40 U/L
TSH	0.78	0.27-4.20 uIU/mL
D-Dimer	363	0-243 ng/mL DDU
High sensitivity C-Reactive protein	111.00	5.00 mg/L
IL-6	22.8	0.0-13.0 Pg/mL
Troponin T	<0.010	≤0.010 Ng/mL
Cepheid COVID-19 PCR	Positive	Negative

**Figure 4:**
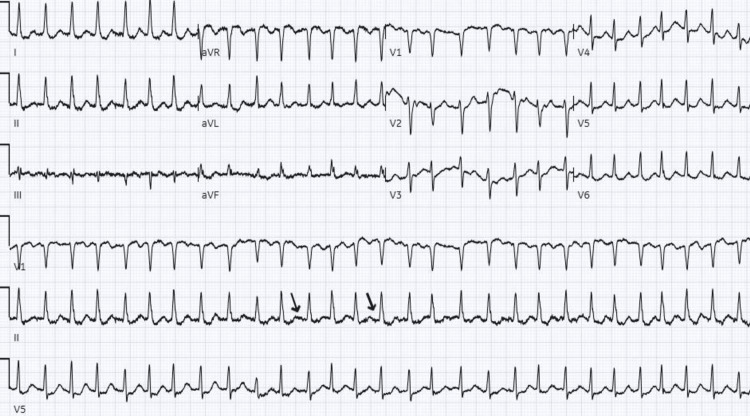
EKG on the sixth day of admission showed an irregularly irregular rhythm with the absence of P waves (black arrows) and a ventricular rate of 175 beats per minute. The findings are suggestive of atrial fibrillation with a rapid ventricular response.

**Figure 5:**
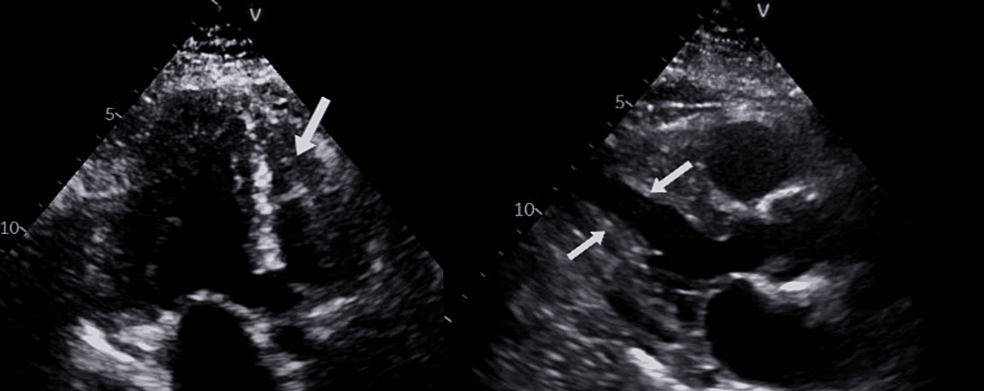
Echocardiogram findings (white arrows) suggestive of mildly increased left ventricular wall thickness with preserved left ventricular ejection fraction (LVEF) and a normal right ventricular systolic function.

On day six of admission, the patient developed tachycardia to 160 beats per minute and complained of palpitations. EKG showed atrial fibrillation with rapid ventricular response (Figure 4). The patient was given metoprolol 2.5mg intravenous push, which did not resolve her symptoms. A 5mg intravenous push of diltiazem also failed to resolve the patient’s symptoms or convert the patient's rhythm to sinus rhythm; following this the patient was started on carvedilol 30mg every six hours, which helped to achieve good rate control. Her CHA2DS2-VASc (congestive heart failure, hypertension, age ≥75 (doubled), diabetes, stroke (doubled), vascular disease, age 65 to 74, and sex category) score was 4 (age, sex, hypertension, and type 2 diabetes mellitus) therefore a shared decision was made to start oral anticoagulation with apixaban 5mg twice daily. Transthoracic echocardiogram (TTE) showed preserved left ventricular ejection fraction without any other abnormality (Figure 5).

The patient improved clinically during her 11-day stay in the hospital but still required supplemental oxygen and, hence, was discharged on home supplemental oxygen. The patient was also discharged on apixaban 5mg oral every 12 hours and diltiazem 120mg oral daily with follow-up to cardiology clinic as an outpatient for newly-diagnosed atrial fibrillation.

## Discussion

SARS-CoV-2 is a single-stranded RNA virus that interacts with the ACE-2 receptor via receptor-mediated endocytosis to invade the host cells [6]. The tissues of the lungs, heart, kidneys, gastrointestinal tract, vasculature, and skin incorporate localized ACE-2 receptors for maintaining their physiological processes [7]. The expression of ACE-2 receptors contributes to the progression of myocardial injury in patients with COVID-19 [8]. The multifactorial etiology of COVID-19 also attributes to cardiovascular complications including myocardial infarction, arrhythmias, and myocarditis potentiated by inflammatory cytokines and troponin elevation [9,10]. In addition, COVID-19 infection is unlikely to cause myocardial fibrosis based on its limited duration of 14-15 days. However, preexisting myocardial fibrosis potentiates the progression of COVID-19 in predisposed patients [11]. The recent studies do not thoroughly elaborate on a possible clinical correlation between atrial fibrillation and COVID-19 infection. However, the putative factors include acid-base imbalances/electrolyte abnormalities, endothelial damage, cytokine storm, and decreased ACE-2 availability. The deterioration of baroreflex triggers the sympathetic drive that increases the risk of atrial fibrillation in patients with COVID-19 infection [3]. Other factors that induce atrial fibrillation in COVID-19 patients include systemic inflammation, microthrombi, myocardial injury, hypoxia, dehydration, drug-drug interaction, drug-induced QT prolongation, coronary spasm, plaque rupture, and increased adrenergic drive. The comorbidities that impact the recovery and prognosis in patients with COVID-19-induced atrial fibrillation include diabetes, hypertension, heart failure, kidney impairment, and older age [12]. These preexisting risk factors potentiate the ACE receptor-mediated viral entry coupled with host systemic inflammatory responses that trigger atrial fibrillation in COVID-19 scenarios. The ACE-2 upregulation in COVID-19 patients attributes to the decreased expression followed by endocytosis of ACE-2 receptors on the cell surface. The reduction in ACE-2 levels in COVID-19 patients correlates with cardiac remodeling that triggers the development of atrial fibrillation [13]. 

Radwan and Schwartz reported a case of NOAF in a 37-year-old male patient with COVID-19 with no significant past medical history or cardiovascular risk factors [14]. The patient presented with left foot pain following a direct trauma with normal vital signs and insignificant laboratory results. The EKG demonstrated an irregularly irregular rhythm suggestive of NOAF. The radiological investigation confirmed a left fifth proximal phalanx displaced fracture requiring an open reduction internal fixation. However, the reverse transcription-polymerase chain reaction (RT-PCR) test preoperatively confirmed a positive result. The patient did not undergo an echocardiogram based on the CHA2DS2-VASc score of 0, which negated the risk of stroke. 

Harhay et al. [15] reported a 90-year-old African American male in ED with a respiratory rate of 24 breaths/mins, heart rate of 140 beats/mins, blood pressure of 141/78mmHg, and temperature of 97.9F. The physical examination confirmed bibasilar crackles, irregularly irregular rhythm, and tachycardia without lower limb edema or jugular venous distention. The initial EKG confirmed a rapid ventricular response with atrial fibrillation. The chest x-ray indicated hazy opacities and diffuse pulmonary edema. In addition, numerous ground-glass opacities were confirmed from the CT chest without IV contrast. Three consecutive blood samples revealed a negative troponin. A grade-1 diastolic dysfunction with a 65-70% ejection fraction was confirmed by an echocardiogram. 

Taha et al. reported two cases of NOAF in patients with a positive COVID-19 result [16]. The first case included a 53-year-old white male with sudden onset palpitations, fatigue, and dyspnea on exertion. The physical examination revealed a body mass index of 30.7kg/m2, temperature of 37.3°C, oxygen saturation (SPO2) of 96%, respiratory rate of 16 breaths/mins, blood pressure of 134/90mmHg, and pulse rate of 136 beats/minute. The 12-lead EKG confirmed an abnormal R-wave progression and pulse rate of 134 bpm with atrial fibrillation. In addition, the D-dimer and the N-terminal (NT)-prohormone brain natriuretic peptide (NT-proBNP) were reported as 0.57mcg/ml (reference range: <0.050mcg/ml) and 1.834pg/dl (reference range: 1-25pg/dl) respectively. The CT angiogram of the chest was unremarkable. The treatment with intravenous diltiazem (25mg) in two divided doses reduced the pulse rate from 99 beats per minute to 90 beats per minute. The oral diltiazem (30mg) was subsequently administered. A normal left ventricular/atrial size, LVEF (60%), and moderate left ventricular hypertrophy were revealed by a subsequent echocardiogram. The patient developed tachycardia (130 beats per minute) on ambulation, dyspnea on exertion, and fatigue after diltiazem dose titration. The transesophageal echocardiogram finding determined the need for cardioversion; however, the patient developed intense fatigue and fever (38.6°C) in the evening. The patient improved dramatically and achieved sinus rhythm after receiving antipyretic Tylenol in special isolation. 

The second case included a 56-year-old Hispanic male who require ED treatment for palpitations and malaise [16]. He also had a history of essential hypertension. He had previously reported to urgent care with frequent palpitations, headaches, night sweats, chills, low-grade fever, and dry cough for one week. The patient was recommended for home isolation and symptomatic treatment after testing positive for COVID-19 infection. The ED assessment confirmed left lower lobe crackles and pulse rate of 144 beats per minute with irregular tachycardia. The EKG revealed left ventricular hypertrophy (LVH) features with a pulse rate of 131 beats per minute, consistent with atrial fibrillation. The chest x-ray confirmed right middle lobe/patchy left perihilar ground-glass airspace disease and left lower lobe infiltrates. The laboratory results indicated lymphopenia, while other findings including hemoglobin A1c, troponin, thyroid-stimulating hormone, complete metabolic panel, and complete blood count did not reveal any abnormality. The patient’s pulse rate improved by 100 beats per minute after receiving normal saline IV fluid bolus (500mL) and IV diltiazem (10mg bolus).

Temgoua et al. reported the case of paroxysmal atrial fibrillation with moderate COVID-19 infection in a middle-aged man with no significant cardiovascular risk factors [17]. A 50-year-old man underwent ED admission for dyspnea on exertion and irregular palpitations. The patient developed myalgia, asthenia, and anorexia one week before ED presentation. The clinical assessment confirmed SPO2 of 87% and mild respiratory distress. In addition, rapid ventricular response and atrial fibrillation were indicated by a resting EKG. COVID-19 was confirmed by an RT-PCR test. The lab assessment confirmed a high C-reactive protein (CRP) of 34.7mg/L, D‐dimer elevation of 585mcg/L, NT-proBNP elevation of 838pg/mL, mild hypoxemia (partial pressure of oxygen (PO2) = 65mm Hg on blood gas analysis), platelet count of 1 72,000/mm3), and hemoglobin of 17g/dL). The chest x-ray did not reveal any significant result, while preserved ejection fraction of 60% was confirmed by TTE. The medical management relied on verapamil (20 mg per 24 hours), rivaroxaban (20mg per 24 hours), IV dexamethasone (6mg per 24 hours/days), and supplemental oxygen. The 24 hours maintenance therapy for normal rhythm was undertaken with flecainide (100mg) following the confirmation of a sinus regular rhythm on the second day of admission. One week's follow-up reaffirmed the sinus rhythm in the asymptomatic patient, which led to the discontinuation of flecainide. 

Seecheran et al. [18] reported a 46-year-old Afro-Caribbean man who required ED treatment for shortness of breath, cough, and fever. The clinical assessment indicated SPO2 in room air of 88%, respiratory rate of 28 breaths per minute, heart rate of 142 beats/minute, and systolic blood pressure of 140mmHg. The physical examination ruled out peripheral edema and confirmed scattered bilateral crackles, normal jugular venous pulse, tachypnea, tachycardia, and hypertension. The 12-lead EKG confirmed ST-T segment changes, atrioventricular block (2:1), and atrial flutter. No acute cardiopulmonary disease was evident on a chest x-ray. No regional wall motion abnormalities and a preserved left ventricular ejection fraction (LVEF) were recorded on a 2D TTE. The laboratory assessments revealed a normal troponin I, creatine kinase-myocardial band (CK-MB), cardiac biomarkers, and D-dimer. The findings, however, indicated a marked elevation in NT proBNP (413 pg/mL). In addition, alveolar-arterial gradient (17mmHg) and mild hypoxia were confirmed by the arterial blood gas results. The pharmacotherapy included beta-blockade, digoxin bolus, and amiodarone. The subsequently performed cardioversion (100J) was unsuccessful, and the patient developed an irregular heartbeat. The RT-PCR test was positive for COVID-19, which necessitated patient transfer to a different unit for intensive management. The patient received atenolol and amiodarone infusion (1mg/minute) that minimized his oxygen demand and improved the symptomatology. The anticoagulation was postponed based on HAS-BLED (hypertension, abnormal renal/liver function, stroke, bleeding history or predisposition, labile international normalized ratio (INR), elderly, drugs/alcohol concomitantly) score (0) and CHADS-VASc score (0) since the patient achieved a normal sinus rhythm. The patient required weekly cardiac assessments via Holter monitor, follow-up visits, and low-dose, orally-administered amiodarone therapy twice daily. 

Bouchlarhem et al. reported the case of a 66-year-old male patient with acute respiratory failure and a history of HbA1C (6.3%), diabetes/hypertension (with good control), metformin (850 mg/d) (for seven years), and ACE inhibitory therapies [19]. The physical examination revealed a body mass index of 31.96kg/m2, height of 1.76cm, weight of 99 kg, temperature of 39.7°C, SpO2 of 83%, respiratory rate of 25 breaths/mins, and dyspnea. The patient appeared neurologically and hemodynamically stable on admission. The COVID-19 finding was positive on the RT-PCT test. In addition, the unenhanced CT chest revealed 25-50% pulmonary infestation by COVID-19 antigen manifested with peripheral ground-glass opacities. The blood assessment revealed LDH, CRP, elevated ferritin, and lymphopenia. In addition, the PO2 of 55mmHg and hypoxemia were confirmed by the arterial blood gas assessment. The patient underwent intensive management supported by oxygen therapy and achieved a 93% SPO2 level. The medication management also included proton pump inhibitors (20mg per day), LMWH (8000UI/12H), dexamethasone (6mg per day), zinc (45mg per 12 hours), and vitamin C (2g per day). The arterial blood gas finding revealed 86% SPO2 despite nasal cannula-directed oxygen therapy on the third day of hospital admission. The patient eventually experienced breathing difficulty that manifested with PO2 of 47mmHg and hypoxemia. The chest CT after contrast enhancement revealed >75% pulmonary consolidation with lesions. This condition warranted 16-hour prone decubitus sessions and oxygen administration (with a flow rate of 14l per minute) via a high concentration mask with a 90% SPO2 target. The patient developed blood pressure (134/80 mmHg), heart rate of 186 beats/minute, spontaneous dyspnea, and palpitations on the fifth day of hospital admission. The rapid ventricular response with atrial fibrillation and narrow/irregular QRS complex tachycardia devoid of P waves were indicated by a subsequent EKG. The continuous infusion of amiodarone following the first bolus was maintained as per the rhythm control strategy. The patient developed hemodynamic instability and NOAF with the ventricular response (205 beats/minute) after a slowing of heart rate (≥140 beats/minute). In addition, the GCS score (8/15), decreased consciousness, and blood pressure reduction (70/50mmHg) were also reported. The administration of 26J external electric shock failed to restore the patient’s consciousness but helped achieve hemodynamic stability confirmed by normal blood pressure (125/80mmHg) and slowing of heart rate (98 beats/minute). A subsequently performed cerebral MRI confirmed an acute ischemic stroke. In addition, thrombolysis, stroke location, and National Institutes of Health Stroke Scale score (35) were contraindicated based on the consciousness alteration. The patient experienced severe neurovegetative manifestations including, tachycardia, extreme bradycardia, and prolonged apnea. The resuscitation interventions failed after five hours of palpitation onset, while the patient developed shock-refractory ventricular arrhythmia after an unrecoverable cardiac arrest. 

Table 2 gives an overview of the presenting symptoms and management strategies used for the above-discussed cases.

**Table 2:** Overview of presenting symptoms and management strategies used in literature review cases. COVID-19: coronavirus disease 2019

Case	Age/Sex	Presenting symptoms	Treatment given to attain rate control in atrial fibrillation.
Radwan et al. [14]	37 year/male	Pain in foot (fracture of proximal 5^th^ phalanx) No symptoms pertaining to COVID-19 present	No rate control agent required
Harhay et al. [15]	90 year/male	Shortness of breath and altered mental status	No rate control agent required
Taha et al. (1) [16]	53 year/male	Dyspnea, fatigue, palpitations	IV diltiazem
Taha et al. (2) [16]	56 year/male	Palpitations, malaise	IV diltiazem
Temagoua et al. [17]	50 year/male	Myalgia, anorexia, asthenia	Oral verapamil
Seecharan et al. [18]	46 year/male	Shortness of breath, cough, fever	Atenolol, amiodarone
Bouchlarhem et al. [19]	66 year/male	Shortness of breath	Amiodarone

## Conclusions

The worldwide reporting of arrhythmias (and related cardiovascular complications) during the COVID-19 pandemic warrant the development of evidence-based treatment pathways to reduce the incidence of cardiac deaths. This case report concisely emphasizes the possible mechanisms triggering atrial fibrillation including acid-base imbalances/electrolyte abnormalities, endothelial damage, cytokine storm, decreased ACE-2 availability, sympathetic drive stimulation, systemic inflammation, microthrombi formation, myocardial injury, and increased adrenergic drive in patients with COVID-19 infection. It also highlights the medical management strategies including beta-blockers and calcium channel blockers use, and their impact on recovery and prognosis. Research should further investigate the pathophysiology concerning atrial fibrillation and its correlation with COVID-19 infection. The follow-up measures are conducive to unraveling the treatment responses and long-term outcomes in patients with COVID-19 and atrial fibrillation. 
